# The Efficacy of Downward Counterfactual Thinking for Regulating Emotional Memories in Anxious Individuals

**DOI:** 10.3389/fpsyg.2021.712066

**Published:** 2022-01-04

**Authors:** Natasha Parikh, Felipe De Brigard, Kevin S. LaBar

**Affiliations:** Center for Cognitive Neuroscience, Department of Psychology & Neuroscience, Duke University, Durham, NC, United States

**Keywords:** anxiety, emotion regulation, counterfactual thinking, autobiographical memory, temporal distancing, electromyography

## Abstract

Aversive autobiographical memories sometimes prompt maladaptive emotional responses and contribute to affective dysfunction in anxiety and depression. One way to regulate the impact of such memories is to create a downward counterfactual thought–a mental simulation of how the event could have been worse–to put what occurred in a more positive light. Despite its intuitive appeal, counterfactual thinking has not been systematically studied for its regulatory efficacy. In the current study, we compared the regulatory impact of downward counterfactual thinking, temporal distancing, and memory rehearsal in 54 adult participants representing a spectrum of trait anxiety. Participants recalled regretful experiences and rated them on valence, arousal, regret, and episodic detail. Two to six days later, they created a downward counterfactual of the remembered event, thought of how they might feel about it 10 years from now, or simply rehearsed it. A day later, participants re-rated the phenomenological characteristics of the events. Across all participants, downward counterfactual thinking, temporal distancing, and memory rehearsal were equally effective at reducing negative affect associated with a memory. However, in individuals with higher trait anxiety, downward counterfactual thinking was more effective than rehearsal for reducing regret, and it was as effective as distancing in reducing arousal. We discuss these results in light of the functional theory of counterfactual thinking and suggest that they motivate further investigation into downward counterfactual thinking as a means to intentionally regulate emotional memories in affective disorders.

## Introduction

Thinking about alternative ways in which past events could have occurred – i.e., counterfactual thought (CFT) – is a common cognitive operation. Earlier work on the psychology of CFT tended to focus on the different conditions under which people were more or less likely to generate mental simulations about alternative ways in which past events could have occurred but did not. Studies showed, for instance, that people were more likely to mentally mutate actions that lead to bad outcomes relative to inactions (Kahneman and Tversky, 1982), abnormal relative to normal actions or events (Kahneman and Miller, 1986), and temporally close relative to distant events (Miller and Gunasegaram, 1990), to name just a few (Roese and Olson, 1995; Byrne, 2005; Mandel et al., 2005; De Brigard and Parikh, 2019). The focus was, then, more on *when* people were more likely than not to engage in CFT rather than on *why* they engaged in CFT to begin with. As a result, questions about the affective consequences of CFT received comparatively less attention.

### Counterfactual Thinking: Functions and Affective Consequences

In an attempt to better understand why people engage in CFT, Roese and Olson (1995) offered the first formulation of what is now known as the *functional theory of counterfactual thinking.* Specifically, they suggested that CFT serves two psychological functions: a *preparative* function and an *affective* function, and that the structure of the CFT was associated with the function it performed. *Upward* CFTs, in which one imagines how a past event could have been *better*, were thought to primarily serve a preparative function: imagining achieving an outcome or a goal one failed to reach may help to improve future chances to do so in equivalent or similar circumstances in the future. For example, a basketball player might imagine that they could have made a shot had they positioned their hand differently, so that next time they are presented with a relevantly similar opportunity, they would be better prepared. Indeed, the regret typically associated with upward CFTs was also thought to help motivate future behavioral improvement.

By contrast, *downward* CFTs, in which one imagines how a past event could have been *worse*, was primarily associated with an affective function: mentally simulating a worse outcome than what it actually occurred may make you feel better about your current circumstances. For instance, a car accident survivor may imagine having suffered much more serious injuries in order to make themselves feel better about totaling their vehicle – which is why relief is typically associated with downward CFT. Indeed, the affective consequences of both upward and downward CFT were interpreted in line with the emotional amplification hypothesis (Kahneman and Miller, 1986), according to which upward CFT tends to heighten negative emotions, whereas downward CFT tends to heighten positive ones.

This view, however, was questioned by Markman and McMullen (2003, 2005) in their *Reflection and Evaluation Model* (REM). According to their proposal, CFT can engage one of two psychologically different modes of mental simulation. When mentally simulating an alternative possibility, individuals may focus on the content of the simulated event, and thus their affect would be biased toward the feeling that would have been elicited had the imagined event been real. This *assimilation* effect would prompt individuals to think of themselves as if the imagined content was part of their current self – a process called “inclusion” (Schwarz and Bless, 1992) – leading them to simply *reflect* about it. The basketball player that failed the shot and engaged in upward CFT may decide to just focus on the imagined content, without regard to what actually occurred, and simply relish on the possibility of having scored – a mental experience that would likely elicit joy rather than regret. Conversely, when the car accident survivor simply focuses on the simulated content, they may feel sick to their stomach by the sheer contemplation of being seriously injured, without regard to the fact that they actually aren’t.

This *reflective* mode of engaging in CFT contrasts with the *evaluative* mode, which stems from a *contrastive* (as opposed to assimilation) effect, whereby individuals focus not so much on the simulated content but on the difference between the imagined situation and the situation they are in when imagining. By “excluding” themselves from the imagined self in their CFT, individuals bias their affect away from the ersatz feeling, prompting thus the opposite valence. Thus, it is only when the basketball player evaluates the unobtained outcome against their current state that regret occurs, just as relief ensues when the driver evaluates the imagined catastrophe against the much desirable, current state of having survived, unscathed. In sum, according to the REM, the emotional amplification hypothesis only explores some affective consequences of CFT, as these can vary depending on the psychological mode one adopts when engaging in these kinds of mental simulations.

As a result, the affective consequences of CFT were reconceived in the revised version of the functional theory of CFT (Epstude and Roese, 2008; Roese and Epstude, 2017). Now, according to the revised functional theory, the primary function of CFT is behavioral modification via cognitive processes, whereas the affective consequences of CFT are seen as merely secondary. Nevertheless, the theory allows for the possibility that affective consequences of CFT could contribute to behavioral modification via one of two “pathways.” First, the theory postulates a *content-specific* pathway, according to which the information represented in a CFT directly transfers to possible future situations. For example, the basketball player that failed to make a shot might imagine having moved their hand differently in a particular way and scoring. Later in the game, they may call upon the specific content of this counterfactual simulation to readjust their hand positioning and make the shot.

But the functional theory also postulates a second, *content-neutral* pathway whereby CFT impacts subsequent behavior irrespective of the specific content in the counterfactual simulation: “That is, independent of the specific meaning contained by it, the CFT may activate mental procedures that carry over into subsequent judgments and behavior” (Roese and Epstude, 2017: 10). Unfortunately, the theory is rather thin in its description of the kinds of “mental procedures” that can be activated as a result of CFT and which can bring about behavioral change – although the authors do mention something they call “counterfactual mindset,” generalization and, importantly, affect and motivation. The question is, then, how precisely do affective reactions to CFT bring about behavioral change?

### Affective Change in Counterfactual Thinking: An Emotional Reappraisal Framework

When it comes to the content-specific pathway, the mechanism by which counterfactual thinking impacts behavior at a later time has been relatively well characterized. The idea, in brief, is that the act of mentally simulating a CFT generates a mental content about a possible way in which a particular past outcome could have been achieved. When individuals successfully encode the imagined content, and are able to retrieve it later on, when the appropriate time comes, behavior improves (Schacter et al., 2015). Evidence to this effect has been reported with a variety of tasks, including anagrams (Roese, 1994; Markman et al., 2008), academic tests (Nasco and Marsh, 1999), and landing planes in a flight simulator (Morris and Moore, 2000). However, when it comes to the content-neutral pathway, the mechanism is less well characterized. Specifically, while the evidence supports an emotional repairing role for downward counterfactual thinking at the time of simulation, it is unclear whether such an effect is long lasting and, if so, why. A first objective of the current paper is not only to evaluate the long-lasting affective impact of downward CFT on the subsequent retrieval of a memory of a past negative event, but also to provide a framework from which to understand such an impact.

The proposed framework is to understand the behavioral change brought about through the content-neutral pathway in terms of the process model of emotion regulation as it applies to memory reactivation. The process model of emotion regulation (Gross, 1999) situates counterfactual thinking as a form of cognitively mediated reappraisal that lies along a continuum of antecedent- and response-focused regulatory strategies. Reappraisal helps an individual manage negative emotional experiences by reinterpreting them in a way that alleviates distress. Reappraisal is a core component of many cognitive behavioral therapies and typically has better long-term effectiveness in alleviating negative affect than other strategies, such as emotional suppression (Aldao et al., 2010). Despite being potentially cognitively costly in the moment for high-arousal situations, reappraisal is thought to be beneficial in the long run due to its complex engagement of elaborated semantic processes that motivate the explicit processing, evaluation, and retention of the emotional event itself (Sheppes et al., 2014).

Reappraisal is theoretically divided into two classes of tactics – reinterpretation and distancing – and counterfactual thinking is considered a form of reinterpretation within this framework (Powers and LaBar, 2019). In contrast to reinterpretation, distancing involves mentally simulating a new spatial, temporal, personal, or hypothetical perspective about the event that serves to psychologically distance oneself from the experience (Powers and LaBar, 2019). For instance, one can imagine a stressful event as occurring far away in time or space, thus detaching ourselves emotionally from the event (Bruehlman-Senecal and Ayduk, 2015). This technique is readily applied to autobiographical memories, and although it requires some effort (Sheppes et al., 2009), is effective at modifying affective responses to acute stressors (Bruehlman-Senecal et al., 2016). Although little laboratory work has been done to directly compare the efficacy of different reappraisal techniques across various contexts, a meta-analytic analysis of emotion regulation studies concluded that distancing is particularly effective (Webb et al., 2012).

While there is some evidence indicating potential benefits of counterfactual thinking on emotion, there is also a noticeable dearth of empirical research examining the relative effectiveness of downward CFTs as a reappraisal technique for regulating emotional memories. To our knowledge, only one recent study has explored the long-term emotional effects of CFTs on the memories from which they are derived. In this study, De Brigard et al. (2018) asked participants to create upward CFTs, downward CFTs, or to attentively recall positive and negative memories. Although the authors found that downward CFT mollified negative valence for negative memories, the study had a number of critical limitations. First, they did not compare downward CFTs to another reappraisal-based emotional regulation technique. Second, they used equal numbers of positive and negative memories in a randomized design, making it difficult to isolate whether the effects seen on negative memories are independent of the inclusion of positive memories (Greenwald, 1976; Suls et al., 1998). Finally, since they did not specify a particular kind of negative memory reported by the participants, their results do not tell us whether downward CFT are differentially effective for distinct kinds of negative memories. Thus, to investigate the role of downward CFT as an emotion regulation technique, the current study compares it against a well characterized distancing technique – i.e., temporal distancing – as well as a simple memory rehearsal task. Additionally, the current study confines the evaluated negative memories to recollections of regretful events, not only to more precisely evaluate the emotional regulation effects of downward CFT, but also because regretful memories are critical to a second aim of the current study, which targets the role of anxiety.

### Maladaptive Counterfactual Thinking in Anxiety

While the functional theory suggests that CFT are adaptive and goal-oriented, it also acknowledges that sometimes these mental simulations are maladaptive and debilitating (Roese and Epstude, 2017). For instance, excessive and ruminative upward CFT has been associated with pathologies such as anxiety, depression, and post-traumatic stress disorder (Nolen-Hoeksema, 2000; Rachman et al., 2000; Roese et al., 2008, 2009). Other studies have shown that, relative to healthy controls, CFTs are phenomenologically different in individuals with anxiety or depression symptomatology (Hajmohammadi and Doost, 2017; Parikh et al., 2020). Critical for our current purposes is research showing a strong association between anxiety (with and without comorbid depression) and rumination of upward CFTs (Lecci et al., 1994; Wrosch et al., 2007). Moreover, repetitive regret-producing upward CFT is a strong predictor of general distress in individuals with anxiety (Roese et al., 2009).

From the point of view of the functional theory of counterfactual thinking, this evidence suggests that anxiety may interfere with the content-specific pathway, turning adaptive upward CFTs into maladaptive ones. But, to our knowledge, no research has focused on the content-neutral pathway in anxiety and, specifically, on the possibility of employing downward CFTs as a technique to emotionally reappraise regret-producing memories and, thus, counteract the pathological rumination of upward CFTs. Though scant, existing research suggests that training in counterfactual reasoning can effectively reduce worry levels across a multi-week training period (Thompson et al., 1999). A somewhat less directed kind of counterfactual thinking appears in therapy as a part of imaginal exposure called *imagery rescripting*, where clients are encouraged to immerse themselves in an imagined alternative scenario to help them process an event that may be too difficult to work with initially. Through this process, the client is eased into working with the memory itself. Case studies, meta-analyses, and a few randomized trials provide evidence for imagery rescripting as effective at reducing symptoms of trauma, social anxiety, depression, nightmares, and other mental ailments (see Arntz, 2012; Morina et al., 2017; Moritz et al., 2018). However, a systematic assessment of downward CFT effectiveness for alleviating negative affect in anxiety and depression is needed. The present study seeks, as a second aim, to evaluate the effectiveness of downward CFT as an emotional reappraisal technique on negative, regret-producing autobiographical memories as a function of individual differences in trait anxiety.

### Design Rationale and Hypotheses

We recruited research participants across a broad range of trait anxiety in a representative community sample and used their index scores as a dimensional measure to correlate with behavioral performance. Our experimental design included several features to improve upon prior literature. We sampled a large number of memories and queried multiple phenomenological characteristics, including affective dimensions of arousal and valence, the intensity of a specific negative emotion (regret), episodic memory detail, and rehearsal frequency. These features were compared pre- and post-regulation to characterize long-term changes in phenomenology. As mentioned above, we used a within-subjects design, comparing downward CFT to both temporal distancing and memory rehearsal as a passive control condition. Since there may be some ameliorate affective benefit in memory rehearsal through habituation mechanisms (Averill et al., 1972), including this condition should help determine which effects are related to the active, cognitively mediated component of regulation that is common across counterfactual thinking and distancing but different from mere rehearsal.

Because downward CFTs should increase negative emotional reactivity during their initial generation (as they involve imagining a worse outcome), we used psychophysiological recordings to validate the experimental manipulation. We chose facial electromyography (EMG) over the corrugator muscle as our primary outcome measure because it is one of the most reliable physiological indices of negative affect generation and emotion regulation (Jackson et al., 2000; Mauss and Robinson, 2009; Wu et al., 2012), although we also collected skin conductance response (SCR) as a secondary outcome measure. Finally, we tested participants’ source memory for the emotion regulation manipulation in the post-regulation testing session by asking them to indicate which technique they were asked to execute for each memory. We also queried participants’ experience with each technique in terms of ease of implementation, their potential future use outside the laboratory, and their subjective feeling of regulatory efficacy.

We hypothesized that, while all regulation techniques might somewhat mitigate subjective affective experience, downward CFTs and temporal distancing should be more effective than rehearsal. Because CFT research has specifically implicated regret as an emotion generated through counterfactual comparison (Petrocelli et al., 2012), we hypothesized that downward counterfactuals would be especially effective in reducing regret. We note that specific emotions like regret are often not sampled during emotion regulation studies, so it is difficult to make predictions regarding how regret would be impacted by distancing or rehearsal. Furthermore, we expected that participants with higher levels of anxiety would not show strong reductions in negative affect after rehearsal compared to those lower in anxiety, given their propensity toward worry and rumination. However, we hypothesized that downward CFTs and distancing would equally benefit high trait-anxious individuals. An exploratory analysis examined whether individual differences in suppression and reappraisal subscales of the Emotion Regulation Questionnaire (ERQ) moderated the relationship between regulation condition and subjective change in affect. Given that downward CFT involves mental simulations of worse experiences, we hypothesized that physiological reactivity would be higher during their generation than during distancing or rehearsal. In an exploratory analysis, we related physiological reactivity changes to subjective changes in affect across conditions.

For our non-affective behavioral measures, we expected source memory for downward CFTs and rehearsal to be better than for distancing, as both counterfactuals and rehearsal involve the reactivation of a unique experienced event (Denny and Ochsner, 2014), in which the former, but not the latter, actively modifies the original information upon reactivation. Conversely, we expected people to report that temporal distancing was easier to use than downward CFTs because it is more generically applied to each memory. Finally, an exploratory analysis examined whether individual differences in suppression and reappraisal subscales of the ERQ moderated the relationship between regulation condition and detail and source accuracy scores.

## Materials and Methods

### Participants

An *a priori* power analysis was completed using R-cran package “pwr2” (Lu et al., 2017) designed for two-way ANOVAs. The analysis used effect size values for session (η^2^_*p*_ = 0.67) and condition (η^2^_*p*_ = 0.17) from the combined valence analysis in De Brigard et al. (2018), an α of 0.05, a β of 0.20, and 100 simulations to result in a goal sample size of 54 participants. During recruitment, participants were prescreened based on four inclusion/exclusion criteria: participants had to be between the ages of 18–39, have no diagnosed psychiatric disorders, take no mood-altering medications, or have any history of neurological damage. Participants who completed and passed the prescreen were asked to participate in our study.

Seventy-four participants completed the prescreen. One participant did not meet our inclusion criteria, 16 were not included due to attrition, 1 chose to discontinue due to discomfort with the procedure, and 2 were unable to continue due to a program malfunction, leaving a total of 54 participants (21 male/33 female, *M*_age_ = 26.59 ± 5.34, *M*_years of education_ = 17.57 ± 2.79, 19 White/Caucasian, 22 Asian, 9 Black/African/African-American, 4 did not specify, 7 also identify as Hispanic/Latinx). Participants were recruited through a community recruitment website and were consented on a protocol approved by Duke University’s Institutional Review Board (#2018-0127). Participants were compensated $12 an hour for their time.

### Procedure

**FIGURE 1:**
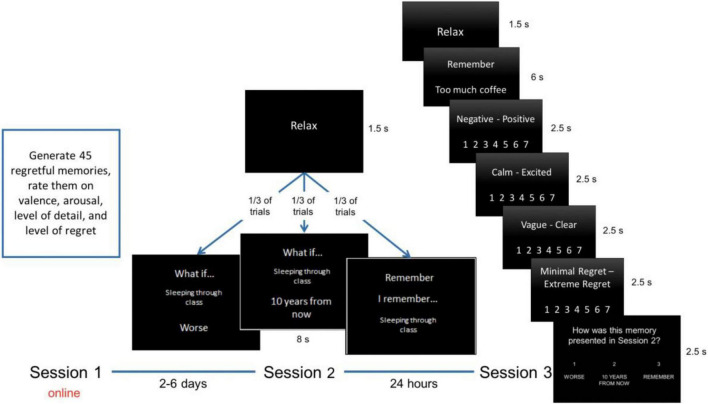
Experimental methods outline. In Session 1, participants listed and rated 45 regretful memories from the past 5 years. In Session 2, participants came into the lab and either created worse alternatives (Downward CFT), imagined how they would feel about the memory in 10 years (Temporal Distancing), or simply recalled details of what occurred (Rehearsal). In Session 3, participants recalled their memories again and re-rated them on various phenomenological measures. They were also asked to recall which technique they had used to simulate the memory on the prior day.

The study involved three sessions (Figure 1). In Session 1, which took place online through Qualtrics, participants reported 45 regretful autobiographical experiences that happened to them in the past 5 years. As they recalled each event, participants were asked to provide a title for the memory (to be used as a retrieval cue in future sessions) and a location and time in which the remembered event occurred. They then rated the memory on their current subjective experience of valence (1-extremely unhappy to 7-extremely happy), arousal (1-extremely calm to 7-extremely excited), regret (1-minimal to 7-extreme), detail of the memory (1-extremely vague to 7- extremely clear), and frequency of rehearsal (1-once a year or less to 7-daily). From these 45 memories, the 30 most negatively valenced memories were included in the experimental set, while the other 15 were set aside for training (9 memories) and for post-experiment measures (6 memories).

Between 2 and 6 days later, participants came into the lab for Session 2 and were trained on each of the conditions used in the experiment: *Temporal Distancing* (i.e., imagine looking back on this memory 10 years from now and think about how it would feel to you then), *Downward Counterfactual* (i.e., imagine how this event could have gone worse), and *Rehearsal* (i.e., simply recall the event naturally). In each condition, participants saw a screen with the memory title and a heading specifying the particular condition. Thus, for memories in the Rehearsal condition, participants saw “Remember” at the top of the screen with their memory’s title underneath. For the other two conditions, corresponding to the regulation techniques, participants saw the words “What if?” followed by the title of the memory. Beneath the memory title, they either saw “Worse” for the Downward Counterfactual condition, or “10 years from now” for the Temporal Distancing condition (see Figure 1).

Participants first practiced each condition in a blocked format. For each condition, participants created the appropriate simulation with two memories in response to generic retrieval cues, e.g., “Whether you exercised in the last 48 h,” followed by one of their actual memories from the training set. In order to confirm the efficacy of the training session, participants were asked to verbally describe what they were simulating during this portion of the training and could not continue on to the next condition unless they accurately described three scenarios in a row for that condition. Additional trials were added until competence was achieved (all participants reached competence with an additional 1 trial per condition). Next, participants practiced the conditions together in a randomized order with the final 6 memories from the training set. Instead of verbally describing their simulations, participants were given 8 s to simulate each condition silently. Furthermore, participants were encouraged to indicate when they finished reading the prompt and began each simulation with a key press.

After completing training, participants were presented with the 30 regretful, negative memories from the experimental set. The memories were randomly assigned to conditions such that baseline valence, regret, and arousal ratings were comparable across conditions per participant, and each condition was presented in a mixed randomized order. The simulation screen indicating the condition and trial was presented for 8 s, and a “Relax” screen was presented for 1.5 s as an intertrial interval (Figure 1).

At the end of Session 2, participants were presented with their 6 final memories (the post-experiment set). These memories were presented with “What if?” but no specific regulation technique. Participants were instructed to use and later report whichever regulation technique (Downward Counterfactual or Temporal Distancing) they felt was most effective at reducing their emotional responses.

Twenty-four hours later, participants returned for Session 3. They were asked to briefly recall the 30 experimental memories again (6 s) and were asked to re-rate them (2.5 s each) on valence (1-unhappy to 7-happy), arousal (1-calm to 7-excited), detail (1-vague to 7-clear), and regret (1-minimal to 7-extreme). They were also asked to answer a source memory question – which technique did you use on this memory during Session 2: “worse,” “10 years from now,” or “remember” – to determine whether a specific manipulation would be better remembered. Again, a 1.5 s “Relax” screen separated trials. At the end of this session, participants were asked whether they found a particular condition to be most effective for regulating their subjective emotional experience, which regulation technique was easier to use, and which they would use (if any) in their daily lives.

### Individual Differences Questionnaires

To understand whether changes in emotional responsiveness were affected by individual differences in participants’ emotion regulation skills, distress, anxiety, or social desirability, all participants completed the Emotion Regulation Questionnaire (ERQ; Gross and John, 2003), Social Desirability Scale (SDS-17; Stöber, 2001), Subjective Units of Distress Scale (SUDS; Wolpe, 1973), and the trait measure of the State-Trait Anxiety Inventory (STAI-Y-2; Spielberger et al., 1983) before they began training in Session 2. We used the reappraisal and suppression subscales from the ERQ to account for individual differences in regulation level prior to experimental training. The SDS-17 was used to test participants’ propensities to provide desired responses as a potential confound to data interpretation. The SUDS was administered at the beginning and end of every session to assess current distress level primarily to ensure that participants did not leave the experimental session in a significantly worse mood state; because most participants scored <20 on the 100-point scale, this scale was not considered further in the analyses. Finally, the STAI trait scale was used as our primary measure of individual differences in trait anxiety.

### Psychophysiology

We measured physiological recordings of skin conductance response (SCR) and facial electromyography (EMG) during the two in-lab sessions. Corrugator EMG activity was our primary outcome measure; it corresponds to a furrowing of the brow and is increased when participants are induced into stressful or aversive states, is correlated with cognitive effort, and is reduced during active down-regulation of negative affect (Van Boxtel and Jessurun, 1993; Sloan, 2004). SCR was taken as a secondary outcome measure, given that SCR is less well-validated as an emotion regulation index but is nonetheless sensitive to sympathetic arousal (Driscoll et al., 2009; Mauss and Robinson, 2009; Matejka et al., 2013). We collected SCR from the palm of the non-dominant hand and facial electromyography from the corrugator supercilii muscle above the right eyebrow. Participants were asked to wash their hands with soap and dry them completely before application of the SCR electrodes. Two Ag-AgCl electrodes with 11 mm diameter contact areas (BIOPAC Systems; Goleta, CA) were placed on the hypothenar eminence of the participant’s non-dominant palm. K-Y Jelly (Reckitt Benckiser; Slough, United Kingdom) was used as a conductive gel. The raw electrodermal signal was sampled at a frequency of 1 kHz and gain amplified at 10 μS/V. A 1-Hz high-pass filter was applied through AcqKnowledge software (BIOPAC Systems; Goleta, CA, United States). Trough-to-peak measurements were extracted using the automated scoring system Autonomate (Green et al., 2014) such that SCR peaks beginning within a second of image onset up through 4 s post-stimulus offset were considered valid responses.

In preparation for the facial electromyography (EMG) collection, we used an alcohol wipe to clean the area above participants’ right eyebrow, used Nuprep Skin Prep (Weaver and Company; Aurora, CO, United States) gel to lightly exfoliate the skin, cleaned this area again with alcohol, and allowed an isotonic 0.05 molar NaCl electrode paste (GEL101; BIOPAC Systems) to soak into the skin for at least a minute before wiping excess off with a facial tissue. Corrugator activity was collected using 2 Ag-AgCl electrodes with a 4 mm diameter contact area (BIOPAC Systems; Goleta, CA, United States) applied directly above the medial edge of the right eyebrow. GEL101 was used as the conductive gel. The two electrodes were placed approximately 1 cm horizontally apart from center to center. The raw EMG signal was sampled at a frequency of 1 kHz. During acquisition using AcqKnowledge software, the signal was high passed at 1 Hz and low passed at 500 Hz, and a notch filter of 60 Hz was applied along with a 100 Hz high pass filter. Post-collection, EMG data was pre-processed in AcqKnowledge by extracting the amplitudes of each data point (taking the absolute value), applying a low pass filter of 16 Hz, downsampling to 15.625 samples/second, and mean value smoothing with a smoothing factor of eight. Per stimulus, EMG values were averaged for 1.5 s prior to stimulus presentation and for the 8 s of stimulus presentation. An EMG difference score was calculated for each stimulus by taking the post-stimulus average and subtracting out the pre-stimulus average to get a baselined mean amplitude value per memory.

### Data Cleaning and Analysis

Although we asked participants for regretful memories, some provided a few memories that were clearly positive in content and valence rating. Thus, we removed trials of memories that were given an initial valence rating of 4 or above (18.6% of all memories). We also eliminated trials for each analysis where the participant either missed or did not respond to the relevant rating question (10.1% of trials for valence, 3.1% for arousal, 2.4% for detail, and 5.5% for regret). These processes did not eliminate any participants from our analyses.

In the current study, we focused on electrophysiological data acquired during the manipulation (Session 2). Since SCR data from that session were highly skewed toward zero (most participants yielded no measurable SCR, perhaps due to effective regulation use), these data could not be analyzed due to low data counts. As such, we only report statistical results from the EMG data taken during the regulation session. Some outliers were identified in the EMG data after creation of our base analysis (effect of condition on EMG scores). Most EMG values clustered between −1 and 1 mV (1st quartile = −0.04 mV, 3rd quartile = 0.18 mV), but a few outliers heavily skewed the data (full range = −52.46 to 125.33 mV). Thus, we cleaned the data by removing values from our base model with residuals over 2.5 standard deviations from the mean. This process removed 32 trials, or around 2.43% of the data. We then re-ran our base model (and all future EMG models) with this cleaned dataset.

To prepare the behavioral data for analysis, difference scores were calculated by taking Session 3 ratings of valence, arousal, detail, and regret and subtracting out the corresponding Session 1 rating for each memory. Using linear and general mixed effects models (LMEMs) in the statistical package R (R Core Team, 2021) with package lme4 (Bates et al., 2015), and starting with a simple model including only main effects, we built analyses one predictor at a time to examine the effects of condition (Rehearsal, Counterfactual Thinking, Temporal Distancing) and individual difference measures (baseline reappraisal use from the ERQ, baseline suppression use from the ERQ, and trait anxiety level from the STAI-Y2) on each behavioral rating. We also added interaction terms between each individual difference measure and condition. Social desirability (SDS-17) scores were included in these analyses as a covariate. We note that SDS-17 scores did not correlate with STAI (*r* = −0.17, *p* = 0.2166), ERQ-reappraisal (*r* = 0.05, *p* = 0.7231) or ERQ-suppression (*r* = −0.12, *p* = 0.3830) scores. Results were corrected for multiple hypotheses testing using Holm’s method, and confidence intervals for LMEMs were calculated using 1000 bootstraps. Pairwise comparisons and multiple regressions with the same predictor variable were corrected for multiple comparisons testing using Holm’s method.

Additionally, we sought to find differences in psychophysiological responses across conditions, and how these responses may have affected behavioral measures (see Supplementary Information for these analyses). For these tests, we used LMEMs similar to the ones described above. Lastly, to determine if participants accurately assessed which techniques were most effective and easy for them to use, we compared participants’ answers on the three summary questions at the end of Session 3. For this analysis, we created subject-level averages per rating per condition, and we ran repeated measures ANOVAs (rmANOVAs) to gauge whether participants’ perceptions of each technique predicted changes in ratings across the experiment, using condition as our repeated measure. Three participants were unable to complete end questionnaires, so only 51 participants were included in these analyses. Pairwise tests were corrected for multiple comparisons testing using Tukey’s HSD (Tukey, 1949).

## Results

### Behavioral Findings

We found no significant effects of simulation condition on arousal, χ^2^(2) = 1.23, *p* = 0.541, valence scores, χ^2^(2) = 3.68, *p* = 0.158, or regret scores, χ^2^(2) = 5.66, *p* = 0.059 (Table 1). As such, we next investigated the interaction terms and individual difference measures for each one of these ratings, which are detailed, in turn, below.

**TABLE 1:** Average values (and standard deviations) of each behavioral rating, separated by simulation condition.

Downward CFT		Temporal distancing	Rehearsal
Valence	0.61 (1.17)	0.76 (1.33)	0.68 (1.26)
Arousal	−0.33 (1.83)	−0.48 (1.84)	−0.47 (1.81)
Regret	−1.13 (1.65)	−1.39 (1.60)	−1.21 (1.64)
Detail	−0.30 (1.55)	−0.25 (1.62)	−0.25 (1.69)
Accuracy	0.45 (0.50)	0.42 (0.49)	0.38 (0.49)

*For valence, arousal, regret, and detail, the values shown indicate the mean change in the rating from Session 1 to Session 3, where increases over time have positive values and decreases over time have negative values. Accuracy of source judgments taken at Session 3 for each memory’s prior regulation condition assignment during Session 2 are presented as mean percent correct.*

#### Arousal

**FIGURE 2:**
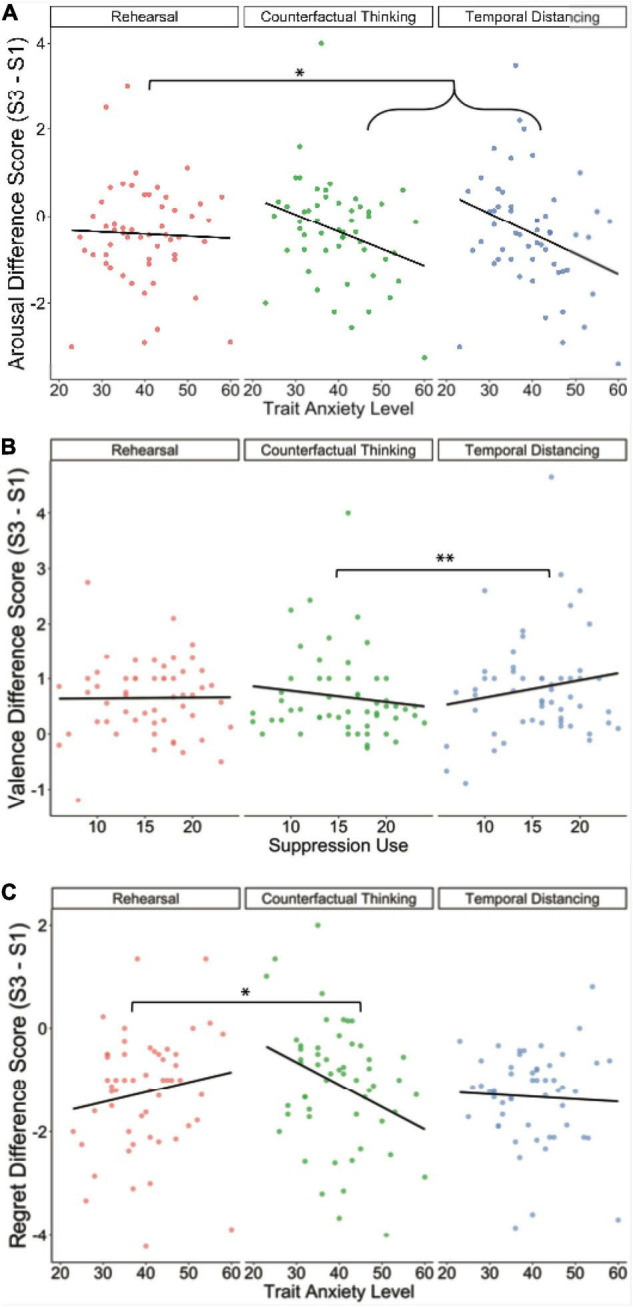
Effects of individual difference measures on behavioral ratings. For ease of display, we have plotted average values per participant here, instead of individual memory values. **(A)** Change in subjective arousal across the experiment by regulation condition and trait anxiety. Higher-anxious individuals showed more arousal reduction in their memories after implementing both active regulation strategies than those with lower anxiety. **(B)** Valence ratings as a function of suppression usage. Compared to those who do not use suppression, participants with higher suppression usage benefited more from temporal distancing but benefited less from CFT in terms of negative valence reduction over time. **(C)** Regret ratings as a function of participants’ trait anxiety. Compared to low-anxious individuals, higher-anxious individuals showed a greater benefit in regret reduction over time following CFT than rehearsal. S1 = Session 1, S3 = Session 3, *p < 0.05, ^**^p < 0.01.

Arousal reductions across the experiment were greater overall in individuals with higher anxiety, χ^2^(1) = 4.18, *p* = 0.041, but this effect interacted with regulation condition, χ^2^(2) = 12.77, *p* = 0.002. Compared to rehearsal, both CFT and distancing yielded a greater reduction in arousal across the experimental sessions in individuals with higher anxiety, *b* = −0.009, *SE* = 0.004, *t*(1222) = −2.47, *p* = 0.014, 95% CI [−0.018, −0.002] (Figure 2A), although the two active regulation conditions did not differ from one another in this regard, *b* = 0.009, *SE* = 0.001, *t*(1224) = 1.28, *p* = 0.202, 95% CI [−0.005, 0.023]. In an exploratory analysis, we also examined individual differences in ERQ subscale scores as moderators, but these predictors showed no significant interactions with condition (see Table 2 for full statistics).

**TABLE 2:** Statistical outputs for each of the linear mixed effects model builds.

Valence	AIC	BIC	c^2^(df)	*p*-value	Detail	AIC	BIC	c^2^(df)	*p*-value
condition	3813.1	3838.5	3.68 (2)	0.158	condition	4799.2	4825.0	0.42 (2)	0.809
anxiety	3816.5	3852	0.58 (1)	0.446	anxiety	4800.3	4836.4	1.09 (1)	0.297
reappraisal	3817.9	3858.5	0.60 (1)	0.439	reappraisal	4801.9	4843.2	0.39 (1)	0.531
suppression	3819.9	3865.6	0.01 (1)	0.941	suppression	4803	4849.5	0.87 (1)	0.350
cond × anxiety	3822.3	3878.2	1.54 (2)	0.463	cond × anxiety	4806.2	4862.9	0.86 (2)	0.652
cond × reapp	3822.1	3888.1	4.21 (2)	0.122	cond × reapp	4805.4	4872.5	4.79 (2)	0.091
cond × supp	3816.8	3893	9.23 (2)	0.010*	cond × supp	4807.7	4885.1	1.72 (2)	0.424
**Arousal**	**AIC**	**BIC**	**c^2^(df)**	***p*-value**	**Source accuracy**	**AIC**	**BIC**	**c^2^(df)**	***p*-value**
condition	4868.0	4893.7	1.23 (2)	0.541	condition	1778.5	1799.2	5.83 (2)	0.054
anxiety	4867.8	4903.8	0.03 (1)	0.966	anxiety	1781.1	1812.2	0.26 (1)	0.613
reappraisal	4869.7	4910.9	4.18 (1)	0.041*	reappraisal	1782.9	1819.2	0.22 (1)	0.638
suppression	4871.4	4917.8	0.03 (1)	0.863	suppression	1784.6	1826.1	0.27 (1)	0.604
cond × anxiety	4862.7	4919.3	12.77 (2)	0.002**	cond × anxiety	1783.2	1835	5.49 (2)	0.064
cond × reapp	4864.2	4931.2	2.42 (2)	0.299	cond × reapp	1782.5	1844.7	4.63 (2)	0.099
cond × supp	4868.0	4945.2	0.27 (2)	0.873	cond × supp	1784.8	1857.3	1.76 (2)	0.414
**Regret**	**AIC**	**BIC**	**c^2^(df)**	***p*-value**					
condition	4572.7	4598.3	5.66 (2)	0.059					
anxiety	4573.7	4609.6	0.94 (1)	0.331					
reappraisal	4574.2	4615.2	1.48 (1)	0.224					
suppression	4571.6	4617.8	4.55 (1)	0.033*					
cond × anxiety	4569.6	4626	6.05 (2)	0.049*					
cond × reapp	4571.9	4638.6	1.65 (2)	0.438					
cond × supp	4571.7	4648.6	4.29 (2)	0.117					

Each table summarizes a different model, and each row in the table shows the iterative addition of regressors to the model from top to bottom and its associated Akaike and Bayesian Information Criterion (AIC and BIC, respectively). Note that the first row, associated with the main effect of condition, displays the main effect model in comparison to a baseline with just an intercept, while each following row is compared to the model in the row above. The chi square, degrees of freedom (df) and associated p-value indicate where the particular conditions are significantly contributing to the model, *p < 0.05, **p < 0.01.

#### Valence

Valence change across the experiment did not differ by anxiety, χ^2^(1) = 0.58, *p* = 0.446, and the interaction of anxiety and regulation condition was not significant (see Table 2). In an exploratory analysis, we found that baseline reappraisal usage from the ERQ, χ^2^(2) = 0.60, *p* = 0.439, and its interaction with regulation condition were non-significant (see Table 2); however, baseline suppression usage from the ERQ did impact valence differentially by regulation condition, χ^2^(2) = 9.30, *p* = 0.010. Neither of the active regulation techniques were different from rehearsal in terms of their impact of suppression use, *b* = −0.001, *SE* = 0.005, *t*(1138) = −0.23, *p* = 0.819, 95% CI [−0.012, 0.011]. However, valence change between distancing and CFT was significantly different, *b* = −0.029, *SE* = 0.009, *t*(1142) = −3.05, *p* = 0.002, 95% CI [−0.046, −0.010], with distancing being positively related to suppression use, *r* = 0.12, but negatively related to CFT, *r* = −0.08 (Figure 2B). Participants with high suppression usage thus benefited from temporal distancing more so than those who did not use suppression, but they did not show as strong of a valence improvement from CFT compared to participants who did not use suppression.

#### Regret

Regret reductions across the experiment showed an interaction between anxiety and regulation condition, χ^2^(2) = 6.05, *p* = 0.049. The impact of anxiety on CFT vs. rehearsal was significantly different, *b* = 0.035, *SE* = 0.013, *t*(1199) = 1.21, *p* = 0.025, 95% CI [0.009, 0.061], such that higher-anxiety participants exhibited an enhanced reduction of regret compared to lower-anxiety participants selectively after using downward CFT (Figure 2C). The impact of anxiety was not significantly different between distancing and rehearsal, *b* = −0.019, *SE* = 0.013, *t*(1199) = −1.46, *p* = 0.289, 95% CI [−0.045, 0.008], nor between distancing and CFT, *b* = 0.016, *SE* = 0.013, *t*(1199) = 1.21, *p* = 0.289, 95% CI [−0.011, 0.042]. In an exploratory analysis, regret was not influenced by baseline reappraisal usage from the ERQ either as a main effect nor as an interaction across regulation conditions (see Table 2); however, regret was impacted by baseline suppression usage, χ^2^(1) = 4.55, *p* = 0.033, where higher suppression use corresponded with less regret reduction across all conditions, *b* = 0.069, *SE* = 0.027, *t*(95.61) = 2.60, *p* = 0.011, 95% CI [0.014, 0.123]. See Table 2 for the full reporting of all regret results.

### Psychophysiology Findings

**FIGURE 3:**
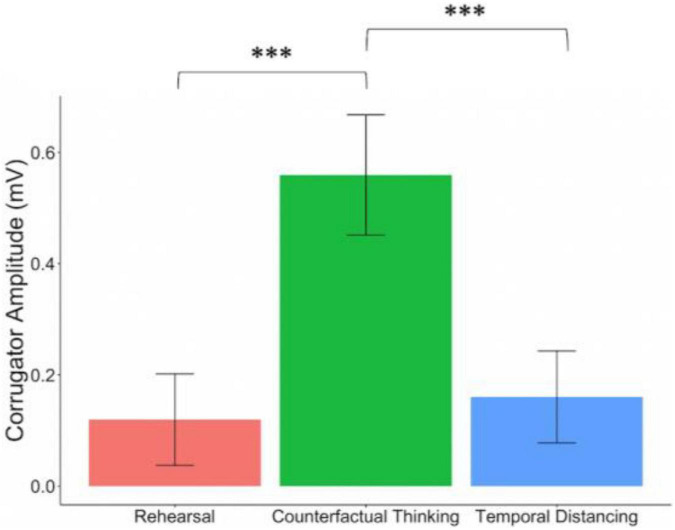
Corrugator EMG amplitude during emotion regulation. Average corrugator response per condition during memory-reactivated emotion regulation (Session 2). Corrugator reactivity was higher during counterfactual thinking than during rehearsal or temporal distancing. Error bars represent standard error of the mean, ^***^p < = 0.001.

Corrugator EMG amplitude significantly differed across regulation conditions, χ^2^(2) = 15.92, *p* < 0.001, with greater corrugator EMG response during CFT relative to both rehearsal, *b* = 0.437, *SE* = 0.124, *t*(1232.56) = 3.53, *p* < 0.001, 95% CI [0.200, 0.691], and distancing, *b* = 0.414, *SE* = 0.122, *t*(1234.07) = 3.40, *p* = 0.001, 95% CI [0.174, 0.675] (Figure 3). Consistent with the hypothesized mechanism of negative affect generation during downward CFT comparisons, which initially involve imagining a worse outcome, these physiological reactivity results validate the experimental manipulation. Further exploratory analyses on how corrugator EMG amplitude related to our phenomenological ratings are available in the Supplementary Information.

### Cognitive Judgment Findings

**FIGURE 4:**
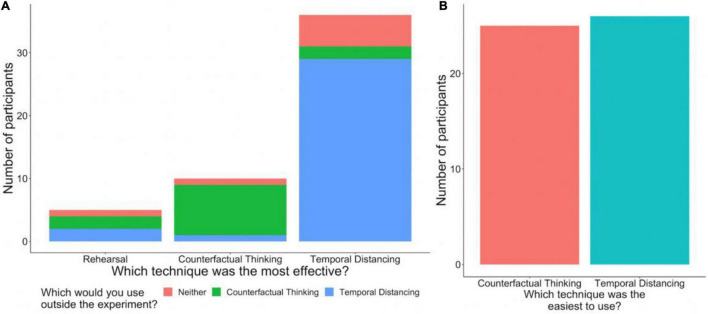
Counts of people choosing each technique for post-experiment questions. **(A)** The number of participants that selected each technique as the most effective, color coded by which technique that participant said they would use in their daily lives. For the most part, individuals’ answers for the two questions were the same. **(B)** The number of participants that chose each technique for which technique was easiest to implement.

Source accuracy and level of detail of the memories were not significantly different across regulation conditions (see Table 1). While most participants reported that temporal distancing was the most effective technique for them and that they would use temporal distancing most often outside of the experiment, they were nearly equally split on which technique they felt was easier to use (Figure 4). In an exploratory analysis, we investigated whether anxiety or ERQ subscales correlated with source accuracy and detail judgments, but no significant effects were found (see Table 2). Further exploratory analyses relating meta-cognitive judgments about effectiveness, ease-of-use, and use outside the experimental setting to objective assessments of affect change, source memory accuracy, and detail judgments are presented in the Supplemental Information.

## Discussion

In this study, participants reactivated regretful autobiographical memories and mentally simulated how they could have gone worse, imagined how they would feel about the remembered event 10 years from now, or simply recalled what occurred without mentally modifying the remembered content. We tested several hypotheses regarding the effectiveness of downward CFT when implemented in specific ways as an intentional emotion regulation technique and assessed its relative benefits to the other techniques across a spectrum of trait anxiety. In what follows, we discuss our findings as they pertain to the hypotheses listed in the introduction.

### Is Counterfactual Thought Effective for Regulating Emotional Memories?

First, to explore the effectiveness of downward CFT as an emotional reappraisal technique for regretful autobiographical memories, we compared it against a “gold standard” reappraisal technique of temporal distancing as well as a baseline of memory rehearsal. We found no support for our initial hypothesis that downward CFT and temporal distancing would be more effective in reducing the strength of negative emotional responses to memories as compared to simple rehearsal. This result was surprising, given that prior work has shown temporal distancing to be more effective in reducing emotional responses than passively evaluating a stimulus (Ahmed et al., 2018). However, previous studies examining the efficacy of temporal distancing have often had participants focus on a single, salient stressful autobiographical memory to regulate (Bruehlman-Senecal and Ayduk, 2015; Bruehlman-Senecal et al., 2016) or have used new negative scenarios that were introduced to the participant during the study (Ahmed et al., 2018). It is possible, then, that the memories used in the current study were either not stressful enough [our average baseline arousal ratings were at the midpoint of our scale, whereas average baseline ratings in Ahmed et al. (2018), were at 73% of their Likert scale] or had already been regulated prior to the experiment from repeated recall and habituation (Averill et al., 1972), and thus did not require as much regulation during the experiment. Furthermore, there is some evidence that participants who learn to use distancing in an experiment also show generalized affect reduction to unregulated stimuli due to possible carryover effects (Denny and Ochsner, 2014). As such, further studies employing memories that have not been previously rehearsed may help to clarify these issues.

Our results also failed to reveal any advantages of CFT in reducing regret relative to both rehearsal and temporal distancing. As mentioned, there is little research on regretful memories specifically, so our hypothesis was rather tentative. Nevertheless, our results suggest that there don’t seem to be an evident advantage in using downward CFT as an emotional regulation technique to reduce levels of regret for regretful autobiographical memories when levels of trait anxiety are not taken into account. The results, however, suggest a different picture when differences in trait anxiety levels are considered.

### Is Counterfactual Thought Sensitive to Individual Differences in Anxiety and Regulatory Style?

Although all three regulatory techniques were successful in altering subjective emotions as a main effect, differences across them emerged when considering individual differences in trait anxiety as a moderator variable. Consistent with our initial hypothesis, participants who reported higher levels of trait anxiety symptomatology displayed stronger reductions in emotional arousal when using CFT and distancing than when simply rehearsing a memory. We also reasoned that if downward CFT serves to repair mood (Markman and McMullen, 2003), high-anxious individuals may have the most room to benefit from its intentional application to quell negative memories, which are characteristically dysregulated in anxiety (Hofmann et al., 2012). Simple memory rehearsal may not be effective in anxious individuals if their typical mode of event recall is accompanied by negative biases. Consistent with this idea, our results indicate that participants with higher levels of trait anxiety reported reduced regret compared to low-anxious individuals when using CFT, although we note that this effect was only statistically significant when compared to rehearsal and not to distancing.

It is interesting that downward CFT could be effective for reducing regret in high-anxious individuals, as regret is typically induced by comparing reality to better alternatives, or upward counterfactual thoughts (Roese and Hur, 1997; Roese and Olson, 1997; Zeelenberg et al., 1998). Our findings suggest that imagining downward counterfactuals, or worse alternatives, may help to counteract the effects of these emotionally maladaptive counterfactual thoughts for people with increased anxiety. However, there may be a limit to the effectiveness of downward CFT, as the counterfactual simulations that induced high corrugator EMG activity were accompanied by reduced increases in valence (see Supplementary Information). These results suggest that while constructing a downward counterfactual may help individuals feel better or less regretful, a highly aversive and/or effortful downward counterfactual simulation period may be counterproductive.

To clarify, it may be worth thinking of this issue in the context of the REM model mentioned before (Markman and McMullen, 2003), according to which the valence elicited by focusing on the simulated counterfactual content alone (reflection) is different from the emotion triggered by contrasting the simulated content with the awareness of one’s own actual situation at the time of simulating the counterfactual (evaluation). In the current study, high corrugator EMG trials may be indicative of participants engaging in reflection, as opposed to evaluation, during the generation of downward CFT, which leads them to just focus on the negative aspects of the imagined alternative (worse) scenario without regard to their current (better) state, thus causing them to stew in the negative emotions of the alternative rather than benefit from the comparison. Manipulating reflection versus evaluation in the context of CFT is not easy, but we think that future studies should compare the relative effectiveness of these two modes of CFT as emotional reappraisal techniques in the context of regretful autobiographical memories in individuals with different levels of trait anxiety.

In an exploratory analysis, we found that the regulatory impact of CFT was also distinctly moderated by participants’ baseline suppression usage. People who tended to push away their emotions and try not to feel them showed larger gains in positive affect from temporal distancing and smaller increases from CFT than those who did not use suppression. This finding may be explained by the nature of how the two techniques theoretically relate to suppression. Distancing is consistent with the goals of suppression, in that distancing allows participants to think about how negative emotions are transient (Bruehlman-Senecal et al., 2016), perhaps assuring people who try to limit emotional expression that these emotions are only temporary. On the other hand, creating a worse counterfactual forces people to confront negative emotions head on, which may be a challenge for people who naturally tend to avoid revealing their emotional reactions.

### Does Corrugator Electromyography Differentiate Downward Counterfactual Thought From Other Techniques?

The functional theory of CFT hypothesizes that generating episodic simulations depicting downward CFT elicits negative affect, but that contrasting the imagined alternative with the actual situation heightens the positive aspects of the latter, ultimately improving one’s feelings about the actual event (Epstude and Roese, 2008). As predicted, our primary objective psychophysiological outcome measure – facial corrugator EMG – was strongest during downward CFT compared to temporal distancing and rehearsal. Corrugator EMG, which corresponds to the furrowing of the eyebrow, is reliably elicited during generation of negative affect, and its activity is reduced during successful emotion regulation (Jackson et al., 2000; Mauss and Robinson, 2009; Wu et al., 2012). Although our results are consistent with this interpretation of corrugator EMG effects, other studies show that corrugator EMG is also responsive to cognitive effort (Van Boxtel and Jessurun, 1993; Sloan, 2004). Because downward CFT requires that a new simulation be generated for each memory, this technique may require more effort than temporal distancing, which can be implemented using the same temporal framing strategy over repeated simulations regardless of the specific details of the memory being regulated (Denny and Ochsner, 2014). To adjudicate between these mechanistic explanations, future research should incorporate trial-by-trial effort and negative affect ratings to correlate with the EMG results and/or manipulate these variables independently during CFT.

### Do the Regulation Strategies Differ on Source Memory Recall and Reported Ease of Use?

We hypothesized that CFT would be the best remembered emotional simulation, but that temporal distancing would be rated as the easiest to use as it requires less tailoring to the specific memory. This hypothesis was not supported by our results; participants did not recall using CFT more accurately than the other two simulation types, nor was temporal distancing rated as easier to use than CFT. Instead, participants rated temporal distancing as the most effective technique at reducing their emotions and as the one they would use most in their daily lives. Crucially, these meta-cognitive judgments were not indicative of true effectiveness, as people’s beliefs did not correlate with how well that technique helped them regulate their emotions objectively (see Supplementary Information). In fact, believing that neither technique was useful to use outside the experiment was perhaps detrimental, as people with that belief showed lower alleviations of regret after CFT manipulation than those who believed in our technique’s ecological validity (see Supplementary Information). We note that these correlational findings are exploratory. Future work should manipulate factors that influence metacognitions about regulation efficacy and show how changes in these perceptions influence emotion regulation objectively.

## Conclusion

In the current study we found some evidence to support theoretical models that suggest downward CFT can be an effective emotion regulation technique in participants with high levels of anxiety. Furthermore, our results suggest that downward CFT may be especially effective in regulating regret associated with a memory in high-anxious individuals relative to merely rehearsing it. Corrugator EMG validated the experimental manipulation but also showed some constraints in the types of memories that could be effectively regulated using generic CFT instructions. Typically, laboratory studies of emotion regulation focus on measuring self-reported affect along the dimensional measures of valence and arousal. Our study demonstrates the added value of measuring the impact of regulation on specific emotions like regret, as different emotion regulation techniques may have more niche applications that remain to be discovered in specific clinical contexts.

Though our results are promising, we note that our study also has its limitations. Our sample was powered for simple main effects between regulation techniques, so further studies with larger sample sizes are needed to replicate our interactions between anxiety and affect change. Additionally, some of the beta estimates of our main findings are relatively small, which may question the applicability of these findings in a clinical population. However, we see this limitation as an opportunity for future research, as we hope further studies would consider exploring the value of CFT as an emotional reappraisal technique for different clinical populations. That being said, it should be noted that our study did not consider other facets of mental health dysfunction (e.g., depression) that may be co-morbid with anxiety and may contribute to the patterns of findings reported here. Because we did not investigate clinically defined populations, future work should extend our results to individuals with specific anxiety and mood disorders. We also note that most participants indicated that temporal distancing was the most desirable technique to use outside of our experiment, although this intuition did not align with objective measures of affect change. Future work is needed to determine whether CFT is an effective technique in more naturalistic settings and for longer periods of time where participants have to implement the technique on their own time.

This study opens the door to further regulation research involving CFT as an intentional means to reappraise emotional memories. Future studies can examine whether downward CFT is best utilized when targeted at regretful memories that are difficult to regulate naturally, and how to optimize its implementation to enhance generalizability. By understanding the set of memories most receptive to counterfactual change, we may also begin to understand the ways in which CFT is maladaptive. With this goal in mind, clinicians and basic scientists can work together to better prevent the debilitating effects of repetitive rumination and obsessive thinking as a risk factor for the development and maintenance of affective disorders.

## Data Availability Statement

The datasets presented in this study can be found in online repositories. The names of the repository/repositories and accession number(s) can be found below: https://github.com/IMC-Lab/RegCFT.

## Ethics Statement

The studies involving human participants were reviewed and approved by the Duke University Institutional Review Board. The patients/participants provided their written informed consent to participate in this study.

## Author Contributions

NP designed the study, conducted the research, analyzed the data, and wrote the manuscript. FD and KL co-supervised NP on all aspects of the project, including consulting on the design, analysis, and interpretation, and writing the manuscript. All authors contributed to the article and approved the submitted version.

## Conflict of Interest

The authors declare that the research was conducted in the absence of any commercial or financial relationships that could be construed as a potential conflict of interest.

## Publisher’s Note

All claims expressed in this article are solely those of the authors and do not necessarily represent those of their affiliated organizations, or those of the publisher, the editors and the reviewers. Any product that may be evaluated in this article, or claim that may be made by its manufacturer, is not guaranteed or endorsed by the publisher.
