# Supplementary material for: The Efficacy of Downward Counterfactual Thinking for Regulating Emotional Memories in Anxious Individuals
Source: Front Psychol. 2022 Jan 4;12:712066. doi: 10.3389/fpsyg.2021.712066 (PMC8764234; doi:10.3389/fpsyg.2021.712066)
Supplement: Supplementary file 1 [file Table_1.DOCX]

**Supplementary Information for:**

**The efficacy of downward counterfactual thinking for regulating emotional memories in anxious individuals**

Natasha Parikh, Felipe De Brigard, & Kevin S. LaBar

Duke University

*Corrugator EMG during emotion regulation as a predictor of behavior change*. Valence change across the experiment was significantly affected by an interaction between EMG amplitude and regulation condition, χ^2^(2) = 9.72, *p* = .039. We used two non-orthogonal contrasts to clarify this interaction. The first contrast found that valence change and EMG amplitude were more positively correlated during rehearsal than during CFT, *b* = 0.098, *SE* = 0.044, *t*(1133.86) = 2.25, *p* = *.*025, 95% CI [0.013, 0.183]. The second contrast found that valence change and corrugator EMG were more positively correlated during distancing than during CFT, *b* = 0.121, *SE* = 0.043, *t*(1114.33) = 2.82, *p* = *.*005, 95% CI [0.032, 0.204] (see Supplementary Figure). Specifically, greater EMG amplitude during CFT correlated with smaller improvements of negative affect across sessions, while greater EMG amplitude during distancing or rehearsal correlated with a greater valence change across sessions. However, we note that these effects are small. EMG amplitudes during emotion regulation had no effect on arousal, regret, or detail changes across the experiment or on source memory for the condition.

*Supplementary Figure:* **Relationship Between Valence Change (Pre-to-Post Regulation) and Corrugator EMG Amplitude During Emotion Regulation.** Positive difference scores reflect more positive valence post- relative to pre-regulation. Each memory is plotted as an individual point with some jitter for display purposes. Note that most corrugator values are around zero, and that the ratings scores are discrete values. Grey areas around the plotted lines indicate standard error around the fit. *p < .05, **p < .01, ***p <= .001.


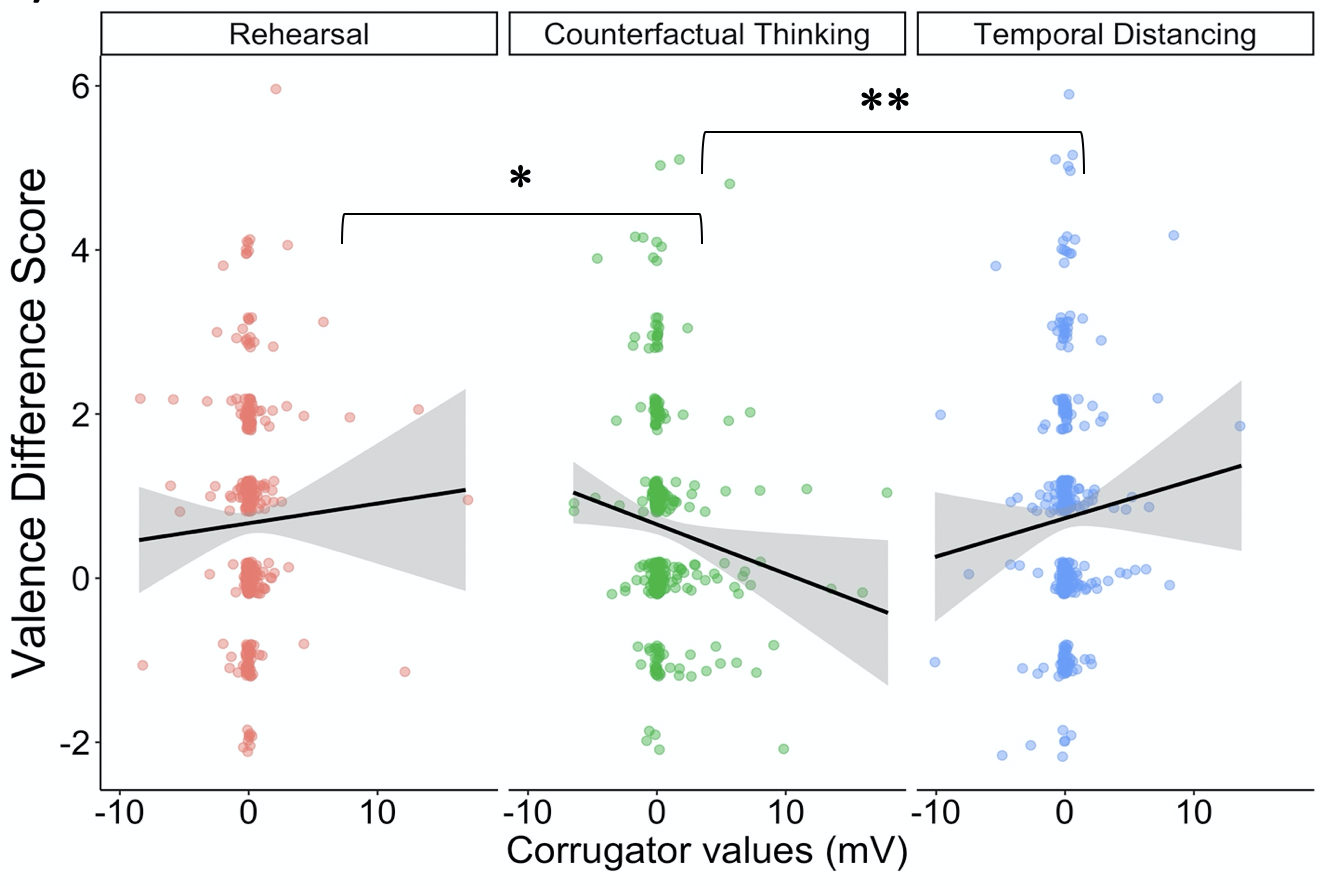


*Relationships between meta-cognitive judgments about regulation abilities and objective measures of affect change and memory phenomenology.* To test whether participants were accurate in their meta-cognitive judgments regarding the effectiveness, ease-of-use, and transfer potential of the regulation strategies outside the experimental setting, we conducted an exploratory repeated measures ANOVA analysis relating these variables to affect changes and memory across regulation conditions. We found a significant interaction of condition and transfer choice (which technique would the participant use outside of the experiment) on regret change across the experiment, *F*(2, 90) = 3.473, *p* = .011, *η^2^_p_* = 0.056. To clarify this interaction, we ran individual ANOVAs within each condition. Transfer choice was significantly related to regret change in the CFT, *F*(2, 45) = 3.891, *p* = .028, *η^2^_p_* = 0.014, but not in the distancing, *F*(2, 45) = 2.825, *p* = .070, *η^2^_p_* = 0.098, or rehearsal, *F*(2, 45) = 0.472, *p* = .021, *η^2^_p_* = 0.627, conditions. Post-hoc tests showed that people who said that they would use distancing outside of the experiment had greater regret change in memories simulated with CFT compared to those who said they would use neither technique outside the experiment, *t*(45) = 2.642, *p* = .030. However, we note that far more participants selected using distancing outside of the experiment than CFT or neither of the techniques, and also that individuals whose regretful feelings benefitted most from CFT scored higher in trait anxiety. Thus this interaction is challenging to interpret. There were no significant effects of post-study questionnaire responses on valence, arousal, detail, or source accuracy (for a summary of all the statistics, see the Supplementary Table).

Supplementary Table

*Statistical Outputs for Each of the Repeated Measures Analyses of Variance (ANOVAs) Conducted on the Behavioral Data Collected at the End of the Study*

|  |  |  |  |  |  |  |  |  | |
| --- | --- | --- | --- | --- | --- | --- | --- | --- | --- |
| **Valence** | F-value (df) | *η^2^_p_* | p-value |  | **Detail** | F-value (df) | *η^2^_p_* | p-value | |
| condition | 0.768 (2) | 0.017 | 0.467 |  | condition | 0.030 (2) | 0.001 | 0.971 | |
| effective | 0.028 (2) | 0.001 | 0.973 |  | effective | 0.248 (2) | 0.011 | 0.781 | |
| easy to use | 0.020 (1) | 0.000 | 0.889 |  | easy to use | 1.134 (1) | 0.025 | 0.293 | |
| will transfer | 0.762 (2) | 0.033 | 0.473 |  | will transfer | 2.079 (2) | 0.085 | 0.137 | |
| cond x effective | 0.942 (4) | 0.04 | 0.443 |  | cond x effective | 0.933 (4) | 0.04 | 0.449 | |
| cond x easy | 0.868 (2) | 0.019 | 0.423 |  | cond x easy | 0.145 (2) | 0.003 | 0.865 | |
| cond x transfer | 1.398 (4) | 0.058 | 0.241 |  | cond x transfer | 0.576 (4) | 0.025 | 0.681 | |
|  |  |  |  |  |  |  |  |  | |
| **Arousal** | F-value (df) | *η^2^_p_* | p-value |  | **Source Accuracy** F-value   (df) | | *η^2^_p_* | | p-value |
| condition | 0.111 (2) | 0.002 | 0.895 |  | condition | 1.699 (2) | 0.036 | 0.189 | |
| effective | 0.439 (2) | 0.019 | 0.647 |  | effective | 0.076 (2) | 0.003 | 0.927 | |
| easy to use | 0.082 (1) | 0.002 | 0.776 |  | easy to use | 0.429 (1) | 0.009 | 0.516 | |
| will transfer | 0.213 (2) | 0.009 | 0.809 |  | will transfer | 3.175 (2) | 0.124 | 0.051 | |
| cond x effective | 0.707 (4) | 0.03 | 0.589 |  | cond x effective | 0.410 (4) | 0.018 | 0.801 | |
| cond x easy | 0.195 (2) | 0.004 | 0.82 |  | cond x easy | 1.248 (2) | 0.027 | 0.292 | |
| cond x transfer | 1.446 (4) | 0.06 | 0.225 |  | cond x transfer | 0.886 (4) | 0.038 | 0.476 | |
|  |  |  |  |  |  |  |  |  | |
| **Regret** | F-value (df) | *η^2^_p_* | p-value |  |  |  |  |  | |
| condition | 1.093 (2) | 0.009 | 0.339 |  |  |  |  |  | |
| effective | 1.442 (2) | 0.031 | 0.247 |  |  |  |  |  | |
| easy to use | 0.573 (1) | 0.006 | 0.453 |  |  |  |  |  | |
| will transfer | 1.476 (2) | 0.032 | 0.239 |  |  |  |  |  | |
| cond x effective | 0.943 (4) | 0.015 | 0.443 |  |  |  |  |  | |
| cond x easy | 0.518 (2) | 0.004 | 0.598 |  |  |  |  |  | |
| cond x transfer | 3.473 (4) | 0.056 | 0.011* |  |  |  |  |  | |

*Note.* Each table summarizes a different model, and each row in the table shows the various results. The F-value, degrees of freedom (df) and associated p-value indicate where the particular conditions are significantly contributing to the model. *p <.05
